# Ringer’s lactate, but not hydroxyethyl starch, prolongs the food intolerance time after major abdominal surgery; an open-labelled clinical trial

**DOI:** 10.1186/s12871-015-0053-5

**Published:** 2015-05-06

**Authors:** Yuhong Li, Rui He, Xiaojiang Ying, Robert G Hahn

**Affiliations:** 1 Department of Anaesthesia, the first Affiliated Hospital, Zhejiang University, Zhejiang, People’s Republic of China; 2 Department of Anaesthesia Shaoxing People’s Hospital, Shaoxing, People’s Republic of China; 3 Department Colorectal Surgery, Shaoxing People’s Hospital, Shaoxing, People’s Republic of China; 4 Research Unit, Södertälje Hospital, and the Section for Anaesthesia, Linköping University, Linköping, Sweden

## Abstract

**Background:**

The infusion of large amounts of Ringer’s lactate prolongs the functional gastrointestinal recovery time and increases the number of complications after open abdominal surgery. We performed an open-labelled clinical trial to determine whether hydroxyethyl starch or Ringer’s lactate exerts these adverse effects when the surgery is performed by laparoscopy.

**Methods:**

Eighty-eight patients scheduled for major abdominal cancer surgery (83% by laparoscopy) received a first-line fluid treatment with 9 ml/kg of either 6% hydroxyethyl starch 130/0.4 (Voluven) or Ringer’s lactate, just after induction of anaesthesia; this was followed by a second-line infusion with 12 ml/kg of either starch or Ringer’s lactate over 1 hour. Further therapy was managed at the discretion of the attending anaesthetist. Outcome data consisted of postoperative gastrointestinal recovery time, complications and length of hospital stay.

**Results:**

The order of the infusions had no impact on the outcome. Both the administration of ≥ 2 L of Ringer’s lactate and the development of a surgical complication were associated with a longer time period of paralytic ileus and food intolerance (two-way ANOVA, *P* < 0.02), but only surgical complications prolonged the length of hospital stay (*P* < 0.001). The independent effect of Ringer’s lactate and complications of food intolerance time amounted to 2 days each. The infusion of ≥ 1 L of hydroxyethyl starch did not adversely affect gastrointestinal recovery.

**Conclusions:**

Ringer’s lactate, but not hydroxyethyl starch, prolonged the gastrointestinal recovery time in patients undergoing laparoscopic cancer surgery. Surgical complications prolonged the hospital stay.

## Background

The choice between colloid and crystalloid fluid for plasma volume support during surgery remains a controversial issue. The current trend to use little (or no) colloid fluid stems from studies in intensive care, where the use of hydroxyethyl starch is associated with an increased risk of acute kidney injury [1,2]. These findings have not been replicated during general surgery [3,4], where starch still has a role in goal-directed fluid therapy, and for the treatment of hypovolaemia [5,6]. Recommendations from the European Medicines Agency hold that the smallest amount possible of starch should be used [7], which implies that the infused volumes of crystalloid might increase.

Colloids carry a risk of allergic reactions, but there are also downsides with crystalloids that may complicate and prolong postoperative care. Infusing more than 3 L of crystalloid [8,9] or 12 ml/kg/hr [10] during open abdominal surgery promotes complications such as prolongation of the gastrointestinal (GI) recovery time. Infusion of about 6 L increases the risk of anastomotic leakage, sepsis, pneumonia and wound infection [11], while infusion of 7 L might be followed by fatal pulmonary oedema [12]. Little is known about dose-dependent adverse effects of colloids except for impairment of coagulation, which is mostly due to excessive dilution of the plasma proteins. However, crystalloid fluid replaces blood loss in the proportion of 3:1 and starch in the proportion of 1:1, which means that larger fluid volumes will be needed to maintain normovolaemia during surgeries if colloid fluids are banned.

The present study explores the incidence of surgical complications, with special reference to GI recovery, and the length of hospital stay when the volumes and the order of infusion of starch and Ringer’s lactate were varied in a cohort of patients undergoing major abdominal or pelvic surgery by the laparoscopic route. Since the study of cholecystectomy by Holte et al. [13], crystalloid programs have been more liberal in laparoscopic abdominal surgery than in open surgery.

## Methods

### Patients

Between July 2011 and March 2013, 88 patients (American Society of Anesthesiologists [ASA] class I or II) with suspected or established gastric, colonic or rectal cancer were recruited to participate in this open-labelled clinical trial. The patients underwent laparoscopic or open GI surgery under combined intravenous and inhalational general anaesthesia. Exclusion criteria were liver or renal dysfunction (liver enzymes > 50% or serum creatinine > 50% of normal), coagulation disturbances, obstructive pulmonary disease, atrial fibrillation and mental disorders.

The protocol was approved by the Ethics Committee of the First Affiliated Hospital, College of Medicine, Zhejiang University (Hangzhou, PR of China; No. 2011150, Official in charge: Zhangfei Shou) and registered at the Chinese Clinical Trial Registry (http://www.chictr.org/en; No. ChiCTR-TNRC-14004479). Written informed consent was obtained from each study subject.

### Procedure

Patients fasted overnight (no solid food or drink), and no premedication was given. Anaesthesia was induced at 8 AM with intravenous lidocaine, fentanyl and TCI-guided propofol. Tracheal intubation was facilitated with cisatracurium (0.2 mg/kg). The patients were mechanically ventilated using a tidal volume of 8 ml/kg, 12 breaths/min and a positive end-expiratory pressure of 3 cm H_2_O. The anaesthesia was maintained with 1–2% of sevoflurane, continuous infusion of propofol (target plasma concentration, 2–3 μg/mL) and/or remifentanil (0.10–0.20 μg/kg/min) and, if needed, intermittent doses of cisatracurium.

Postoperative pain relief was managed by patient-controlled intravenous analgesia (PCA) using sufentanil 100 μg in 100 ml in isotonic saline which was given at a rate of 2 ml/h. Bolus injections were 2 ml using a lockout-time of 5 min and a maximum of 30 ml during 4 hours.

Monitoring consisted of a bispectral index (BIS monitor) where the anaesthesia was guided to reach a BIS value of between 40 and 60. Central haemodynamics were followed via the FloTrac™/Vigileo system and monitoring also included pulse oximetry, electrocardiography and heart rate. Urine was collected via a bladder catheter inserted just after the tracheal intubation. Body temperature was maintained at 35.5°C or higher. Haemodynamic data from the bolus infusions have been published elsewhere [14].

Measurements of blood haemoglobin and the serum concentrations of creatinine, protein, albumin, glutamic pyruvic transaminase and blood urea nitrogen were taken before the surgery and on the first postoperative day.

### Fluid therapy

No fluid was infused during the induction of general anaesthesia. Each patient was allocated to one of four fluid programs consisting of a first-line bolus infusion initiated about 10 min after tracheal intubation, and a second-line continuous infusion initiated when surgery started.

GROUP 1. Bolus with starch, continuous with starch.

GROUP 2. Preloading with Ringer’s lactate, bolus with starch, continuous with Ringer’s lactate.

GROUP 3. Bolus with starch, continuous with Ringer’s lactate.

GROUP 4. Bolus with Ringer’s, continuous with Ringer’s lactate.

The patients were not allocated to the four fluid programs individually but allocated in blocks of 25–30. They reflect the beliefs, based on previous work, that the order of the infusion may affect fluid retention and consequently outcome [15] and that preoperative dehydration can increase the number of postoperative complications [16].

#### Bolus infusions

Three bolus infusions of either 6% hydroxyethyl starch 130/0.4 (Voluven®; Fresenius Kabi Deutschland GmbH, Bad Homburg, Germany) or Ringer's lactate. Each of the boluses were given in the volume of 3 ml/kg over 7 min via an infusion pump (IEC 601–1; Abbott Laboratories, Chicago, IL).

#### Continuous infusions

When surgery was about to begin, a continuous infusion of either starch or Ringer’s lactate was given via an infusion pump at a rate of 12 ml/kg over 1 hour.

Further fluid therapy was given at the discretion of the attending anaesthetist, but the volumes were recorded.

To prevent preoperative dehydration, 30 patients in the starch + Ringer group (GROUP 2) also received preloading with 500 mL of Ringer's lactate as a slow drip over 2 h, beginning 3 h before the induction of anaesthesia.

### Outcome measures

The outcome measures included the time period spent in the postoperative care unit (PACU), time period of paralytic ileus, time to tolerance of oral food, length of hospital stay and postoperative complications. The complications were taken from the medical records from the in-hospital period and interpreted according to the definitions used in the current worldwide International Surgical Outcomes Study (ISOS, see http://isos.org.uk). We considered postoperative complications to include anastomotic leak, surgical site infection, pneumonia, acute respiratory distress syndrome, pulmonary oedema, sepsis, myocardial infarction, cardiac arrhythmias, cardiac arrest, vomiting, severe pain, pulmonary embolism, stroke, postoperative haemorrhage, acute kidney injury and re-operation.

### Statistics

The study was originally powered to differentiate between haemodynamic responses to the post-induction bolus infusion [14]. A *post hoc* analysis showed that the key outcome measure used in the present report, i.e. the difference in food intolerance time between patients who received < 2 L and ≥ 2 L of Ringer’s lactate, was determined at the level of P < 0.05 with a power of 99.9%.

Data are presented as means (SD). Differences between groups were evaluated statistically by a one-, two- or three-way analysis of variance (ANOVA).

Receiver operator characteristic (ROC) curves were used to illustrate the fluid volume needed to predict prolongation of the postoperative period of food intolerance to ≥ 5 days as well as the occurrence of any other complication (IBM SPSS Statistics Version 22).

Significance was defined as *P* < 0.05.

## Results

### Treatment groups

The demographic data and surgical details were similar in the four groups (Table 1, top). Eighty-three per cent of the operations were performed by laparoscopy. No patient was admitted to the ICU after the surgery.

**Table 1:** **Details of patients, surgeries and fluid treatment, selected blood parameters and outcome measures**

Fluid treatment	Group 1	Group 2	Group 3	Group 4	ANOVA
Preloading 500 ml	No	Yes	No	No	
Bolus infusion 9 ml/kg	Starch	Starch	Starch	Ringer	
Continuous infusion 12 ml/kg/1 hr	Starch	Ringer	Ringer	Ringer	
**N**	20	28	18	22	
**Age (years)**	62 (10)	58 (13)	56 (12)	63 (8)	NS
**Body weight (kg)**	59 (7)	59 (8)	62 (8)	58 (8)	NS
**Males (per cent)**	75	54	72	77	
**Gastric/colonic/rectal cancer (N)**	11/2/7	5/9/14	0/7/11	17/3/2	
**Open surgery (N)**	5	4	5	1	
**Anaesthesia time (min)**	262 (96)	272 (62)	269 (60)	242 (65)	NS
**Operating time (min)**	206 (97)	201 (64)	206 (63)	195 (62)	NS
**Blood loss (ml)**	205 (138)	160 (114)	200 (89)	194 (114)	NS
**Urine output (ml)**	516 (402)	557 (168)	441 (286)	361 (156)	NS
**Starch (ml)**	1125 (235)	1018 (94)	1000 (0)	420 (179)	*P* < 0.001
**Ringer’s lactate (ml)**	1475 (472)	1438 (534)	1333 (542)	2295 (480)	P < 0.001
**Erythrocytes (N; ml)**	5; 420 (277)	1; 700	1; 300	6; 454 (282)	
**Plasma (N; ml)**	0	1; 500	1; 230	3; 417 (40)	
**Blood haemoglobin (g/L)**					
**Before surgery**	128 (17)	124 (24)	127 (24)	124 (22)	NS
**After surgery**	112 (15)	115 (15)	113 (19)	111 (16)	NS
**Serum creatinine (μmol/L)**					
**Before surgery**	69 (13)	68 (17)	65 (14)	75 (14)	NS
**After surgery**	63 (12)	61 (18)	58 (12)	64 (10)	NS
**Time in PACU (min)**	71 (25)	60 (23)	66 (34)	97 (40)	*P* < 0.001
**Paralytic ileus (days)**	3.2 (0.9)	3.6 (1.4)	2.7 (0.9)	3.5 (1.1)	*P* = 0.054
**Food intolerance (days)**	4.5 (1.9)	4.6 (1.9)	4.6 (2.4)	6.5 (3.5)	*P* < 0.03
**Complications per operation**	0.35	0.36	0.28	0.23	NS
**Infectious complications (N)**	5	2	3	1	
**Bleeding complications (N)**	1	3	1	1	
**Length of hospital stay (days)**	13.0 (4.6)	13.9 (2.6)	13.3 (3.0)	13.3 (4.7)	NS

Data are the mean (SD) or the actual number (N) of patients. NS = not statistically significant.

Table 1 illustrates that the order of the infusions did not affect the outcome parameters. However, the patients who received Ringer’s lactate, both for the bolus and for the continuous infusion (Group 4), had a longer PACU time and a longer period of time with food intolerance, while the number of postoperative complications and the length of hospital stay were not increased (Table 1, bottom).

The 22 patients who had a postoperative complication stayed longer in hospital and were also slightly older than the others (Table 2).

**Table 2:** **Comparison of operations with and without postoperative complications**

	No complications	Complications	ANOVA
**N**	66	22	
**Age (years)**	58 (12)	64 (9)	*P* < 0.05
**Body weight (kg)**	60 (9)	59 (6)	NS
**Males (per cent)**	71	59	
**Anaesthesia time (min)**	250 (68)	295 (72)	*P* < 0.01
**Operating time (min)**	190 (65)	235 (78)	*P* < 0.01
**Blood loss (ml)**	175 (107)	229 (131)	
**Urine output (ml)**	426 (225)	614 (359)	*P* < 0.02
**Starch (ml)**	860 (328)	1000 (267)	NS
**Ringer’s lactate (ml)**	1655 (681)	1590 (453)	NS
**Erythrocytes (N; ml)**	6; 432 (264)	6; 466 (272)	
**Plasma (N; ml)**	3; 335 (228)	2; 213 (205)	
**Time in PACU (min)**	76 (35)	66 (27)	NS
**Paralytic ileus (days)**	3.1 (1.1)	3.8 (1.3)	*P* < 0.01
**Food intolerance (days)**	4.6 (2.2)	6.2 (3.2)	*P* < 0.03
**Length of hospital stay (days)**	12.7 (3.4)	15.6 (3.9)	*P* < 0.002

Data are the mean (SD) or the actual number (N) of patients. NS = not statistically significant.

### Univariate analyses

A comparison was made between the outcomes of all patients who received < 2 L or ≥ 2 L of Ringer’s lactate, regardless of study group. Those given ≥ 2 L had a longer PACU time and a longer period of time with paralytic ileus and food intolerance, while postoperative complications and the length of hospital stay were not increased (Table 3).

**Table 3:** **Operations with infusion of either < 2 L or ≥ 2 L of Ringer’s lactate**

	Ringer’s lactate <2 L	Ringer’s lactate ≥2 L	ANOVA
**N**	55	33	
**Age (years)**	58 (12)	63 (10)	P < 0.05
**Body weight (kg)**	60 (8)	59 (7)	NS
**Males (per cent)**	67	70	
**Anaesthesia time (min)**	258 (74)	268 (68)	NS
**Operating time (min)**	196 (76)	211 (63)	NS
**Blood loss (ml)**	167 (93)	221 (138)	P < 0.05
**Urine output (ml)**	444 (258)	502 (286)	NS
**Starch (ml)**	1009 (225)	705 (361)	P < 0.001
**Ringer’s lactate (ml)**	1236 (358)	2310 (348)	P < 0.001
**Erythrocytes (N; ml)**	4; 183 (416)	6; 416 (40)	
**Plasma (N; ml)**	6; 367 (175)	7; 518 (308)	
**Time in PACU (min)**	66 (30)	86 (37)	P < 0.01
**Paralytic ileus (days)**	3.1 (1.2)	3.7 (1.1)	P < 0.023
**Food intolerance (days)**	4.4 (2.1)	6.1 (3.0)	P < 0.004
**Complications per operation**	0.27	0.21	NS
**Infectious complications (N)**	7	4	
**Bleeding complications (N)**	3	3	
**Length of hospital stay (days)**	13.3 (3.7)	13.7 (3.9)	NS

Data are the mean (SD) or N = the number of patients. NS = not statistically significant.

The 66 patients who received ≥ 1 L of starch had shorter PACU time (65 *versus* 96 min; *P* < 0.001) and a shorter time of food intolerance (4.6 *versus* 6.5 days, *P* < 0.002). A smaller fraction of these patients developed one or several postoperative complications (13.6% *versus* 28.8%), but this difference did not reach statistical significance (*P* = 0.14).

### Multivariate analyses

A two-way ANOVA confirmed that both Ringer’s lactate ≥ 2 L and complications prolonged the period of paralytic ileus and food intolerance, while only complications prolonged the length of hospital stay (Table 4).

**Table 4:** **Significance levels in two-way ANOVA evaluating simultaneously the effect of administration of 2 L or more of Ringer’s lactate and postoperative surgical complications on various outcome measures in 88 patients**

	Ringer’s lactate ≥2 L	Complications
**Anaesthesia time (min)**	NS	P < 0.03
**Operating time (min)**	NS	P < 0.03
**Blood loss (ml)**	NS	NS
**Urine output (ml)**	NS	P < 0.04
**Time in PACU (min)**	P = 0.05	NS
**Paralytic ileus (days)**	P < 0.02	P < 0.01
**Food intolerance (days)**	P < 0.004	P < 0.007
**Length of hospital stay (days)**	NS	P < 0.001

NS = not statistically significant.

**Figure 1:**
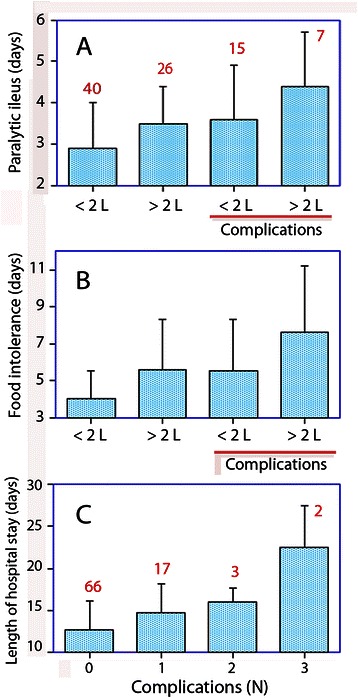
Duration of paralytic ileus **(A)** and food intolerance **(B)** depending on infusion of either < 2 L or ≥ 2 L of Ringer’s lactate during surgery and whether postoperative complications developed. **(C)** The length of hospital stay increased with the number of complications (ANOVA *P* < 0.002). Data are the mean, and the error bars are the standard deviation. The number of patients in each group is indicated in red.

For example, no complications and Ringer’s lactate < 2 L had a mean period of paralytic ileus of 2.9 (1.1) days, and ≥ 2 L was associated with 3.5 (0.9) days. The corresponding time periods for patients *with* complications were 3.6 (1.3) and 4.4 (1.3) days, respectively (Figure 1A). The effect of Ringer’s lactate and complications on the food intolerance time was somewhat greater, amounting to about 2 days each (Figure 1B). No independent effect on PACU time was found.

In contrast, the infusion of ≥ 1 L of starch *reduced* the time period of food intolerance by 2 days (*P* < 0.005) when simultaneously considering the effect of surgical complications (*P* < 0.014; two-way ANOVA).

When testing all three factors simultaneously, only Ringer’s lactate ≥ 2 L and complications prolonged the period of food intolerance (both *P* < 0.02), while the alleviating effect of ≥ 1 L of starch did not reach statistical significance (*P* = 0.17).

### ROC curves

**Figure 2:**
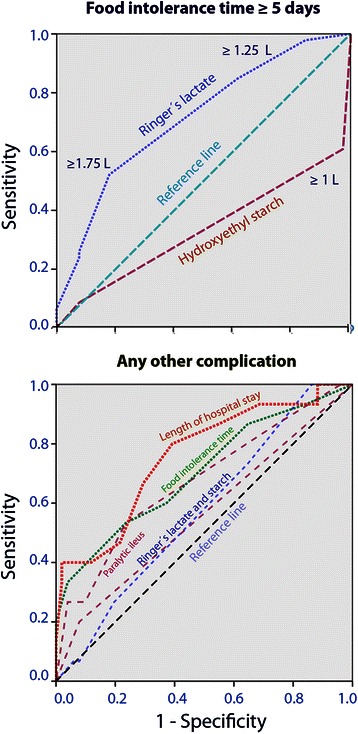
ROC curves illustrating the fluid volume needed **(A)** to predict prolongation of the postoperative period of food intolerance to ≥ 5 days and **(B)** to predict the occurrence of any other complication. Closeness to the reference line implies lack of relationship.

The most appropriate cut-off for the fluid volume that affected the postoperative food intolerance time to ≥ 5 days amounted to ≥ 1.75 L for Ringer’s lactate and ≥ 1 L for starch (Figure 2A). The area under the ROC curves was 0.71 (Ringer’s lactate) and 0.34 (starch, both *P* < 0.05).

Figure 2B illustrates the sensitivity and specificity of perioperative parameters to indicate the occurrence of a surgical complication. The area under the ROC curves was 0.76 (hospital time), 0.70 (food intolerance time) and 0.67 (paralytic ileus, all *P* < 0.05) while the fluids had 0.57 (Ringer’s lactate) and 0.56 (starch, none being statistically significant).

### Exploratory analyses

No statistically significant difference in total urine output was found among the four groups (Table 1). However, the excreted urine divided by the sum of infused starch and Ringer’s lactate was lower in ‘Ringer’s-lactate-only’ Group 4 compared with the pooled data from the three starch groups (20% [SD 11] *versus* 13% [6], *P* < 0.02).

The type of cancer or the use of laparoscopic or open surgery had no statistical correlation with the outcome measures, though there was a trend toward higher urine flow in open surgery (urine flow 2.2 *versus* 1.7 ml/min).

Cardiac output was lower at the end of the bolus infusions in the “Ringer’s-lactate-only” group (3.1 [SD 0.4] L/min *versus* 3.6 [0.7] L/min, *P* < 0.003) but differences had cancelled out at 1 hour of surgery. At that time, the cardiac output for all patients was 4.7 (1.4) L/min, which represented 78% (SD 22) of baseline (i.e. before the induction of anaesthesia). However, at 1 hour, the heart rate was lower compared to baseline in those who had received a combination of both starch and Ringer’s lactate (73% [SD 15] bpm) as compared to those who had received only one of these fluids (87% [SD 17] bpm; P < 0.001; Table 5).

**Table 5:** **Selected haemodynamic parameters measured with FloTrac/Vigileo**

Fluid treatment	Group 1	Group 2	Group 3	Group 4	ANOVA
Preloading 500 ml	No	Yes	No	No	
Bolus infusion 9 ml/kg before surgery	Starch	Starch	Starch	Ringer	
Continuous infusion 12 ml/kg/1 hr	Starch	Ringer	Ringer	Ringer	
**Cardiac output (L/min)**					
Before anaesthesia	5.8 (1.6)	6.1 (1.6)	7.1 (2.2)	6.2 (1.4)	NS
Beginning of surgery	3.5 (0.7)	3.6 (0.6)	3.6 (0.7)	3.1 (0.4)	P< 0.03
1 hour of surgery	5.0 (1.4)	4.5 (1.4)	4.8 (1.6)	4.6 (1.3)	NS
Relative to before anaesthesia (%)					s
After bolus infusions	63 (19)	61 (16)	55 (19)	53 (13)	NS
1 hour of surgery	90 (24)	75 (18)	70 (23)	78 (20)	P< 0.03
**Mean arterial pressure (mmHg)**					
Before anaesthesia	101 (13)	106 (14)	107 (12)	109 (10)	NS
Beginning of surgery	71 (10)	76 (91)	70 (8)	70 (11)	NS
1 hour of surgery	92 (16)	95 (16)	97 (31)	93 (19)	NS
Relative to before anaesthesia (%)					
Beginning of surgery	70 (9)	72 (10)	66 (8)	65 (10)	P< 0.03
1 hour of surgery	92 (16)	90 (16)	92 (31)	86 (19)	NS
**Heart rate (bpm)**					
Before anaesthesia	75 (11)	79 (14)	84 (18)	72 (9)	NS
Beginning of surgery	59 (7)	56 (8)	56 (8)	55 (8)	NS
1 hour of surgery	64 (9)	56 (10)	61 (11)	60 (9)	P< 0.03
Relative to before anaesthesia (%)					
Beginning of surgery	73 (10)	72 (15)	69 (12)	78 (8)	NS
1 hour of surgery	88 (19)	73 (14)	74 (18)	86 (14)	P< 0.002

Data are the mean (SD).

Blood chemistry showed similar values before and after the surgery. The mean decrease in the blood haemoglobin concentration was 9%, total protein and bilirubin 10%, albumin 13% and blood urea nitrogen decreased by 15%, while the glutamic pyruvic transaminase increased by 39%. Serum creatinine decreased by 11% (Table 1) without being statistically related to the amount of starch used.

## Discussion

Infusion of ≥ 2 L of Ringer’s lactate prolonged food intolerance time by 2 days, while infusion of ≥ 1 L of hydroxyethyl starch did not have this effect. Beyond the GI recovery, the fluid volumes used did not correlate with the number of postoperative complications, such as infection and bleeding. However, patients who developed complications had a prolonged period of paralytic ileus and food intolerance that was clearly additive to the effect of ≥ 2 L of Ringer’s lactate.

We hoped to disclose differences in outcome depending on the sequence of infusing these fluids, but found no such effects. Starch given as the first infusion after induction of general anaesthesia yielded virtually identical outcome measures, regardless of whether starch was continued or switched to Ringer’s lactate, or when dehydration had been prevented by slowly infusing 500 ml of Ringer’s lactate in the early morning before surgery (Table 1).

Only when using Ringer’s lactate for both the first and second fluid infusion was there a significant prolongation of the time required for overcoming the surgery-induced paralytic ileus and food intolerance. However, the prolongation actually became apparent in all patient groups when the amount of infused Ringer’s lactate was 2 L. In fact, the ROC curve revealed a clear trend toward a prolongation of the food intolerance time in response to as little as ≥ 1.25 L of Ringer’s lactate (Figure 2A).

Hydroxyethyl starch maintained urinary excretion as well as, or even better than, Ringer’s lactate during these lengthy (mean 4 hrs) laparoscopic surgeries, which is probably due to an improvement of the renal perfusion. Our recordings of central haemodynamics provided no good explanation for this finding. Changes in serum creatinine levels were minimal and did not correlate with the amount of infused starch, which has been an issue [1-4]. Starch also seemed to reduce the duration of paralytic ileus (Figure 2A). However, the multivariate analysis suggested that most of this effect could be explained by a need for a smaller volume of Ringer’s lactate when a large amount of starch was infused.

The negative effect of large crystalloid fluid volumes on outcome after open abdominal surgery has been recognised for more than a decade [8-12,17,18]. A restrictive fluid policy facilitates wound healing and reduces the time required for GI recovery, and sometimes reduces the length of hospital stay. “Zero balance” has even been proposed [19,20]. However, not all of the previous authors found advantages in using a restrictive fluid policy. MacKay *et al.* [21] replicated the pioneering work by Lobo [8] but did not report the same benefits, despite the inclusion of three times as many patients. Kabon *et al.* [22] could not find any difference in wound healing after infusing very large fluid volumes (16–18 ml/kg/hr) compared to smaller volumes (8 ml/kg/hr).

A meta-analysis by Varadhan & Lobo [9] showed that fluid volumes between 1.75 and 2.75 L per day are associated with better overall outcomes. This conclusion is valid for crystalloid fluid during open abdominal surgery, but little is known about the effect of colloid and crystalloid fluid on the outcome after laparoscopic surgery. The present study fills in this gap by showing that ≥ 2 L of Ringer’s lactate significantly prolongs the GI recovery time during laparoscopic cancer surgery, both in patients with and without postoperative complications.

Many authors report slow GI recovery as a complication, which makes it difficult to separate the contributions of fluid-dependent and fluid-independent adverse events to the length of hospital stay [17]. Here, GI recovery was considered separately from other outcome parameters. By doing so, we disclosed clear relationships between fluid volumes and GI recovery but no relationship between fluid volumes and surgical complications like bleeding and infection (Table 2, Figure 2B). However, when evaluating this result, one must consider that excessive amounts of crystalloid or colloid fluid were never used. The largest volume of Ringer’s lactate amounted to 3 L and the largest volume of starch was 1.5 L. Although the volumes used are far from those associated with pulmonary problems [11,12], the benefits of crystalloid fluid restriction can still be extended to laparoscopic surgery, as only 17% of the present operations were performed by an open approach.

Limitations include the fact that the study was an open-labelled trial in which all patients received both fluids. The volumes differed, except for the post-induction bolus rounds and the first hour of surgery when the fluid therapy was strictly standardised. The study was originally powered to differentiate between the haemodynamic responses to the post-induction bolus infusions with starch and Ringer’s lactate [14] although our *post hoc* power analysis showed that valuable information could be obtained about outcome measures as well. Complications were collected from the medical records and not by a prospective survey. The uneven number of patients in the Tables can be explained by the lack of access to the postoperative charts for the first studied 23 patients, who therefore had to be excluded. This problem was due to a change of hospital by the author who organised the data collection (Y.L.).

The incidence of postoperative nausea was low and mild in form, and just like prolonged GI recovery, was not included in the group of surgical complications. We regard the other outcome measures to be trustworthy and valid. The blood sample for analysis of serum creatinine was taken the first day after the surgery, while kidney injury can be more clearly distinguished on the second and third postoperative day. However, if the kidneys are harmed, an increase is still apparent on the first postoperative day [2].

## Conclusions

In 88 patients who underwent major abdominal or pelvic surgery, usually via the laparoscopic route (83%), the use of 6% hydroxyethyl starch 130/0.4 (Voluven) did not prolong the time period required for postoperative GI recovery. In contrast, administration of ≥ 2 L of Ringer’s lactate increased the duration of paralytic ileus by 0.7 days and of food intolerance by 2 days, regardless of whether GI recovery was prolonged by surgical complications.
